# Characterization of the Immune Cell Infiltration Profile in Pancreatic Carcinoma to Aid in Immunotherapy

**DOI:** 10.3389/fonc.2021.677609

**Published:** 2021-05-13

**Authors:** De Luo, Fei Kuang, Juan Du, Mengjia Zhou, Fangyi Peng, Yu Gan, Cheng Fang, Xiaoli Yang, Bo Li, Song Su

**Affiliations:** ^1^ Department of Hepatobiliary Surgery, Affiliated Hospital of Southwest Medical University, Luzhou, China; ^2^ Department of General Surgery, Changhai Hospital of The Second Military Medical University, Shanghai, China; ^3^ Department of Clinical Medicine, Southwest Medical University, Luzhou, China; ^4^ Department of Ultrasound, Seventh People’s Hospital of Shanghai University of Traditional Chinese Medicine, Shanghai, China

**Keywords:** immune cell infiltration, prognostic biomarker, pancreatic carcinoma, tumor microenvironment, immunotherapy

## Abstract

The tumor microenvironment (TME) is comprised of tumor cells, infiltrating immune cells, and stroma. Multiple reports suggest that the immune cell infiltration (ICI) in TME is strongly associated with responsiveness to immunotherapy and prognosis of certain cancers. Thus far, the ICI profile of pancreatic carcinoma (PC) remains unclear. Here, we employed two algorithms to characterize the ICI profile of PC patients. Based on our results, we identified 2 ICI patterns and calculated the ICI score by using principal component analysis. Furthermore, we revealed that patients with low ICI scores had a better prognosis, compared to high ICI scores. Moreover, we discovered that a low tumor mutation burden (TMB) offered better overall survival (OS), relative to high TMB. In this study, a high ICI score referred to elevated PD-L1/TGF-β levels, increased activation of cell cycle pathway and DNA repair pathway, as well as reduced expression of immune-activation-related genes. We also demonstrated that three metabolic pathways were suppressed in the low ICI score group. These data may explain why a high ICI score equates to a poor prognosis. Based on our analysis, the ICI score can be used as an effective predictor of PC prognosis. Hence, establishing an ICI profile, based on a large patient population, will not only enhance our knowledge of TME but also aid in the development of immunotherapies specific to PC.

## Introduction

Pancreatic carcinoma (PC) is an aggressive cancer that provides a 5-year overall survival (OS) rate to <9% of affected patients (1–4). Despite advancements in the field, PC prognosis remains poor, due to late diagnosis and restrictive treatment strategies (1, 3). Immunotherapy is an approach that enhances host immunity, which, in turn, targets and eliminates tumor cells (5–7). It has ushered in new advancements in anti-tumor treatment, including PC therapy, carrying enormous survival benefits (6, 8, 9). However, only a few PC patients are responsive to immunotherapy (8–10). Thus, exploring new predictive biomarkers for PC patients’ prognosis and developing novel therapeutic strategies are of urgent need.

The lack of patient response to immunotherapy likely stems from our limited understanding of the tumor microenvironment (TME). TME is comprised of tumor-infiltrating immune cells (TIICs) and stroma, and it is specific to individual cancers (11). Hence, PC-specific TME has a unique immune cell infiltration (ICI) and characteristically desmoplastic stroma (11, 12). Several studies on TME showed a strong correlation between ICI and tumor growth, metastasis, and sensitivity to immunotherapy (13). One example is tumor-associated macrophages (TAMs), which drive tumorigenesis, *via* the release of immunosuppressive cytokines, like TGF-β, thereby promoting poor prognosis (14, 15). Alternately, elevated levels of CD4^+^ T cells and CD8^+^ T cell infiltration are associated with better prognosis and responsiveness to immunotherapy (16). However, TIICs alone do not regulate sensitivity to immunotherapy. In fact, in some studies, patients with high TIICs were shown to be immunotherapy-resistant, likely due to TIICs dysfunction brought on by TAM-regulated immunosuppressive cytokines (17, 18). In addition, a massive infiltration of stromal cells can further block TIICs infiltration into tumor tissue (19). These data suggest that immunotherapy responsiveness is strongly modulated by intrinsic events in TME and not by the action of an individual cell population. Thus far, there are few reports on an extensive ICI profile in TME of PC patients. In the last few decades, advancements in next-generation sequencing (NGS) technology, particularly the NGS algorithm technology, have enabled the extraction of large amounts of biological data on PC formation and metastasis (20).

Here, we employed two algorithms, CIBERSORT and ESTIMATE, to assess the gene-expression profiles of a large number of tumor samples and establish an extensive overview of the PC immune landscape (21, 22). In addition, we categorized PC patients into two distinct subgroups, based on the ICI patterns. Finally, we established an ICI score to characterize the immune cell infiltration profile of PC, which could accurately predict patient prognosis and be of benefit to the selection of subsequent immunotherapeutic strategies.

## Materials and Methods

**Figure 1:**
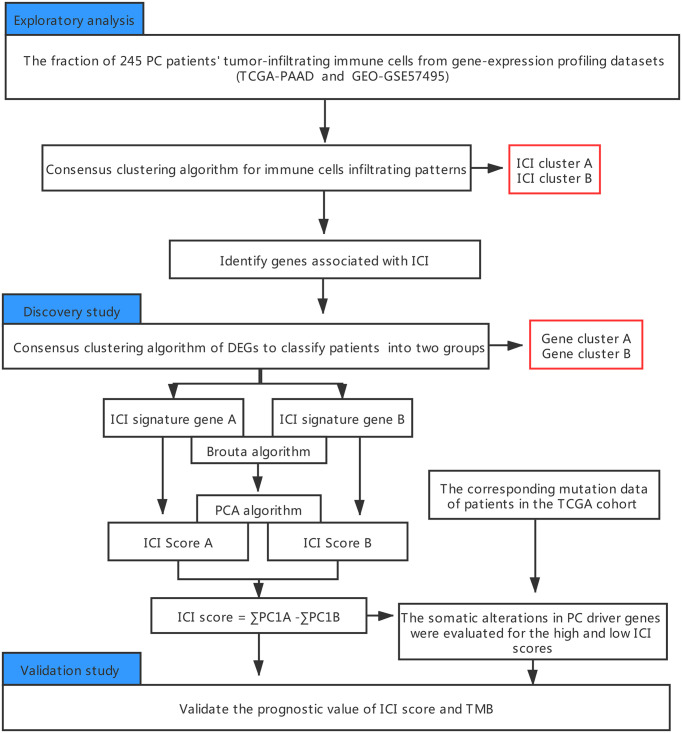
An outline of the research and data collection protocol.

An outline of the study, along with the data collection procedure, is summarized in **Figure 1**.

### PC Datasets and Samples

The datasets analyzed in the current study are available at The Cancer Genome Atlas (TCGA) at https://portal.gdc.cancer.gov, reference number TCGA-PAAD, and Gene Expression Omnibus (GEO) at https://www.ncbi.nlm.nih.gov/geo/, under the accession number GSE57495.

### Consensus Clustering for TIICs

The “CIBERSORT” R package, carrying an LM22 signature and 1000 permutations, was employed for the quantification of individual immune cell infiltrating levels in PC (21). ESTIMATE was used to establish the immune and stromal scores in PC samples (23). The hierarchical agglomerative clustering of PC was completed per ICI pattern for individual PC samples. Subsequently, the unsupervised clustering “Pam” technique, closely following the Euclidean and Ward’s linkage, was employed, using the “ConsensuClusterPlus” R package (24), and replicated 1,000X to verify classification stability.

### Differentially Expressed Genes (DEGs) Related to the ICI Phenotype

Patients were grouped into ICI clusters, according to their ICI profile, to identify genes associated with the ICI patterns. DEGs, among ICI subtypes, were identified by using the “limma” R package, and with the following criteria: *P *< 0.05 and absolute fold-change > 1.4.

### Dimension Reduction and ICI Score Computation

Next, using unsupervised clustering, the patients were stratified, according to their DEG values. The positively- and negatively-regulated DEG values were further classified as ICI signature gene (ICISG) A (ICISG-A) or ICISG-B, respectively. Moreover, the Boruta algorithm was employed for the dimension reduction of ICISG-A and ICISG-B (22), and the principal component 1 was retrieved as the signature score, using PCA. Finally, the ICI scores of individual patients were obtained using a technique similar to the gene expression grade index (25), as described below:

$$ICIscore=\Sigma PC1_{A}-\Sigma PC1_{B}.$$

### Somatic Data Collection

First, we downloaded the TCGA cohort’s genetic information from the TCGA data portal. Next, we calculated the sum of non-synonymous mutations in PC. The somatic changes, in PC driver genes, were next assessed against high and low ICI scores. The PC driver genes were recognized using the “maftool” R package (26). Lastly, the leading 20 driver DEGs were chosen for further analysis.

### Statistical Analyses

GraphPad Prism version7.0 or SPSS version 21.0 (IBM Corporation, Armonk, NY, USA) was employed for all statistical analyses. Comparison between groups was performed with the Wilcoxon test. The X-tile software, which iteratively selects potential threshold to maximize rank statistic, was employed for PC patient classification into subgroups and to decrease computational batch outcome (27). The Kaplan-Meier plotter produced the OS curves for each examined category. The log-rank test analyzed significance. The chi-square test examined the association between the ICI scores and somatic mutation frequency. The Spearman analysis calculated the correlation coefficient. Two-tailed *P*< 0.05 was the statistical significance threshold.

## Results

### The ICI Profile in PC TME

245 tumor samples were obtained from the TCGA-PAAD and GEO-GSE57495 cohorts. Infiltrating immune cells were quantified in PC tissues, using both CIBERSORT and ESTIMATE algorithms. Next, these patients were categorized into discrete subgroups, *via* unsupervised clustering, using the “ConsesusClusterPlus” package of the R software.

**Figure 2:**
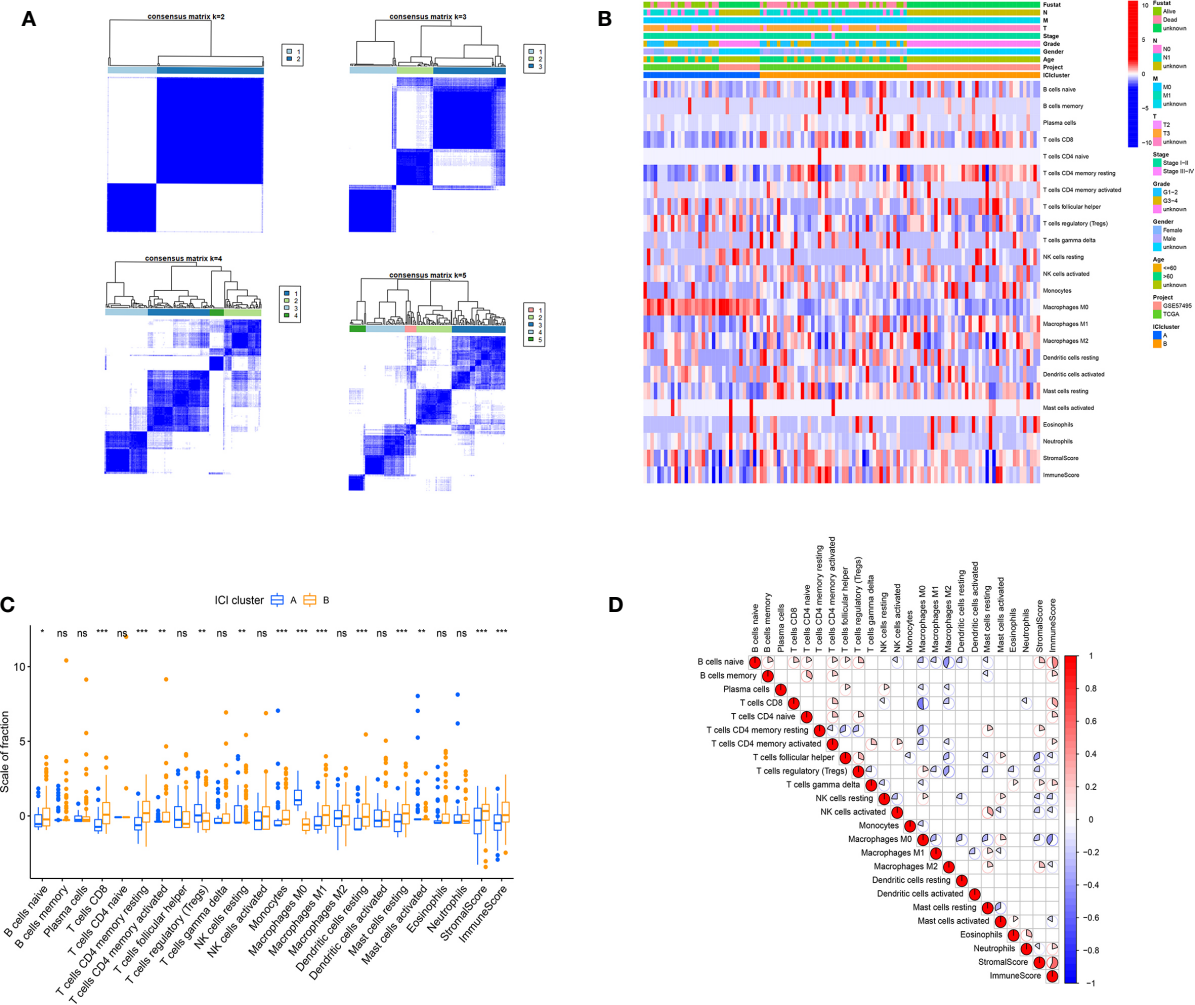
The ICI profile in PC TME. **(A)** Consensus matrixes of PC samples, representing each k (k=2–5). **(B)** Unsupervised clustering of TIICs in PC cohorts. Rows denote TIICs and columns denote PC sample. **(C)** TIICs fractions in both ICI clusters. Significance computed using the Wilcoxon test, *P < 0.05; **P < 0.01; ***P < 0.001. ns, not significant. **(D)** Cellular interaction of the TIICs types.

Based on our analysis, two isolated ICI subtypes were found (**Figures 2A, B**). To delineate the intrinsic changes that lead to the distinct phenotypes, we further analyzed the ICI pattern of PC TME. As depicted in **Figure 2C**, ICI cluster A had a large population of regulatory T cells, M0 macrophages, and activated mast cells infiltration, whereas ICI cluster B had more naive B cells, CD8^+^ T cells, resting memory CD4^+^ T cells, activated memory CD4^+^ T cells, resting NK cells, monocytes, M1 macrophages, resting dendritic cells, and resting mast cells. Additionally, the ICI cluster B phenotype also exhibited elevated immune and stromal scores. We, next, compiled the correlation coefficient heatmap to illustrate the ICI profile in PC TME (**Figure 2D**).

### Identification of the Immune Genetics Subtype

**Figure 3:**
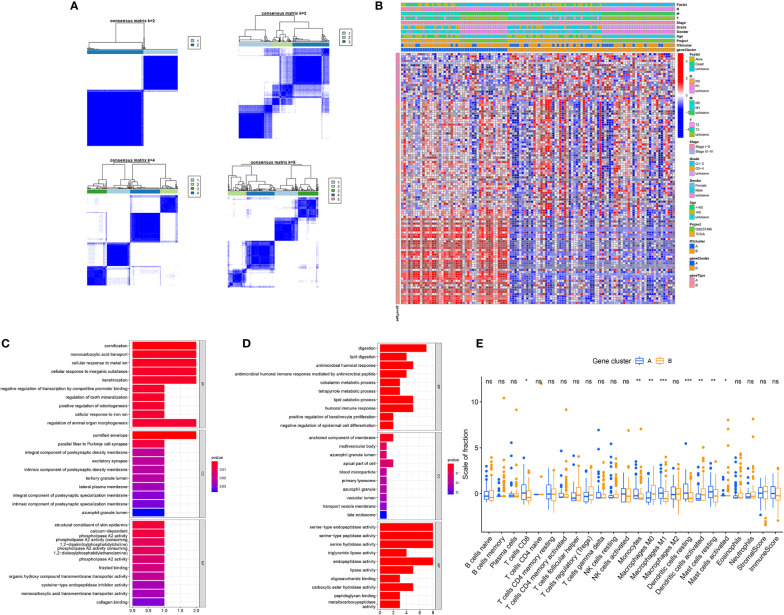
Identification of Immunogenic Gene Subtypes. **(A)** Consensus matrixes of PC samples, representing each k (k=2–5). **(B)** Unsupervised clustering of common DEGs in both ICI cluster cohorts for patient stratification: gene clusters A and B. **(C, D)** Gene Ontology (GO) enrichment analysis of two ICISGs: ICISG-A **(C)** and ICISG-B **(D)**. The x-axis represents gene quantity in each GO term. **(E)** The fraction of TIICs in both gene clusters. Plots of the immune and stromal score of both gene clusters. Significance was computed using the Wilcoxon test, *P < 0.05; **P < 0.01; ***P < 0.001. ns, not significant.

To elucidate the genetic features of discrete immune-related phenotypes, we conducted genetic differential analyses, using the “limma” program of R software. Using unsupervised clustering of 114 DEGs (obtained from prior differential analyses), we categorized the TCGA and GEO cohorts into two genomic clusters, namely gene clusters A and B (**Figure 3A**). Among the 114 DEGs, the 22 genes that were positively correlated with the gene cluster in PC patients were placed in the ICISG-A category and the remaining DEGs were placed in the ICISG-B category. Simultaneously, to eliminate noise and the presence of repetitive genes, we employed the Boruta algorithm to conduct dimension reduction in ICISG-A and ICISG-B. We, also, generated a heatmap illustrating the gene expression profile of 114 DEGs within ICISG-A and ICISG-B (**Figure 3B**), with the “clusterProfiler” R program. As illustrated in **Figures 3C, D**, we summarized significantly enriched biological processes in both ICISG-A and ICISG-B, using the Gene Ontology (GO) enrichment analysis. Furthermore, gene cluster A contained a large population of CD8^+^ T cells, monocytes, M1 macrophages, resting dendritic cells, and resting mast cells infiltration. Alternately, gene cluster B had massive infiltration of M0 macrophages, activated dendritic cells, and activated mast cells (**Figure 3E**).

### ICI Score Computation and Association Between ICI Score and Somatic Variation

**Figure 4:**
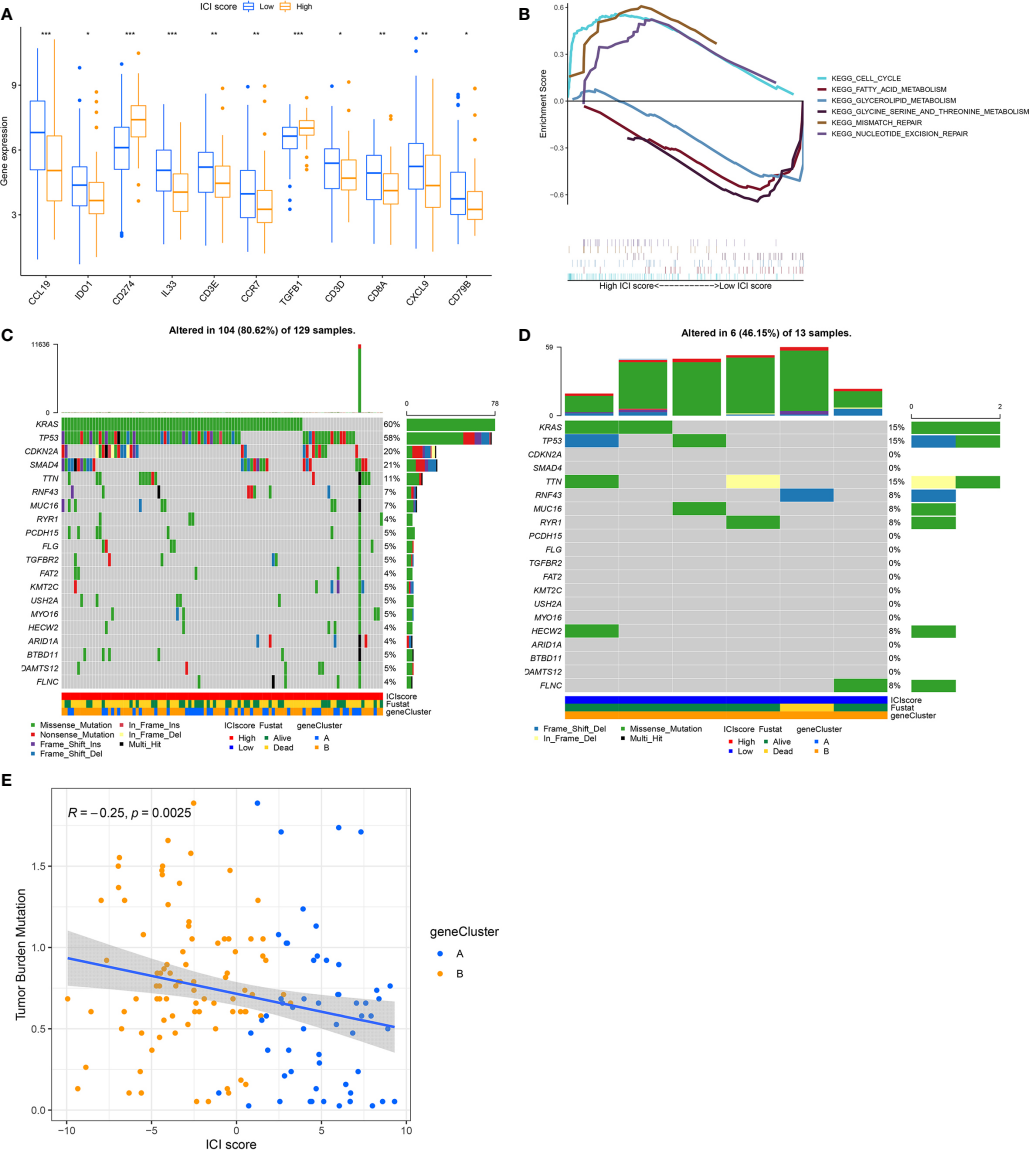
The ICI Score Generation and Association between the ICI Scores and Somatic Variants. **(A)** Evaluation of the immune-checkpoint-related genes and immune-activation-related genes in high and low ICI score subgroups. Significance was computed using the Wilcoxon test, *P < 0.05; **P < 0.01; ***P < 0.001. **(B)** Enrichment plots reveal that the cell cycle and DNA repair pathways were significantly enriched and activated in the high ICI score group, relative to the low ICI group. Conversely, three metabolism signaling pathways (glycerolipid metabolism; fatty acid metabolism; glycine, serine and threonine metabolism) were significantly enriched and inhibited in the low ICI score group, relative to the high ICI group. **(C, D)** The oncoPrint was compiled, based on a high ICI score **(C)** and low ICI score **(D)**. Each column represents a single patient. **(E)** Scatter plots illustrating a negative association between the ICI score and mutation load in the TCGA cohort. The Spearman correlation between ICI scores and mutation load is also provided (*P* = 0.0025).

To establish quantitative indicators for the ICI profile, we employed the principal-component analysis (PCA) to calculate 2 cluster scores: (1) the ICI score A from ICISG-A and (2) the ICI score B from ICISG-B. The scores of ICI score A and ICI score B were calculated for individual patients, based on a compilation of related scores. Subsequently, we obtained the ICI score, which was used as an estimator of PC prognosis. Patients in the TCGA and GEO cohorts were then classified into 2 categories, according to an optimal ICI score threshold, produced by the X-tile software. To assess the immunological activities and drug sensitivity of each group, we chose for evaluation CD274 (PD-L1) and TGFB1 (TGF-β) as the immune checkpoint-related genes, and CCL19, CCR7, CD3D, CD3E, CD79B, IL33, CD8A, CXCL9, and IDO1 as immune-activation-related genes. Accordingly, we demonstrated that immune-activation-related genes were markedly upregulated in the low ICI score group, relative to the high ICI score group (**Figure 4A**). Additionally, the high ICI group exhibited increased levels of TGF-β and PD-L1 relative to the low ICI score group, as evidenced by the Wilcoxon test (**Figure 4A**). In addition, the gene set enrichment analysis (GSEA) revealed that the cell cycle and DNA repair pathways, particularly, mismatch repair and nucleotide excision repair, were significantly enriched and activated in the high ICI score group, as opposed to the low ICI score group. Conversely, three metabolic pathways (glycerolipid metabolism; fatty acid metabolism; glycine, serine and threonine metabolism) were significantly enriched and inhibited in the low ICI score group (**Figure 4B**).

Previous evidence revealed that tumors with a high tumor burden mutation (TMB, non-synonymous variants) contributed to an elevated number of infiltrating CD8^+^ T cells that target and destroy tumors. Based on this, TMB may be an influencing factor in patient prognosis and responsiveness to cancer immunotherapy (28, 29). The KEYNOTE 012 clinical trial showed that a high TMB was correlated with increased sensitivity to PD-1 blockades and a favorable prognosis in PC patients (30). Here, we evaluated somatic PC driver gene variants’ distribution in low and high ICI score subgroups, using “maftools”. The top 20 DEGs were then selected for further analysis (**Figures 4C, D**). We demonstrated that the levels of KRAS, TP53, CDKN2A, and SMAD4 were markedly altered in the low and high ICI score subgroups (chi-square test, *P*<0.05). Given the clinical significance of TMB, we, next, examined the intrinsic relationship between the TMB and ICI score to assess genetic imprints of individual ICI score subgroups. As illustrated in **Figure 4E**, our correlation analysis validated that the ICI score was negatively associated with TMB (Spearman coefficient: R = -0.25, *P* = 0.0025).

### The Significance of ICI Score in PC Prognosis

**Figure 5:**
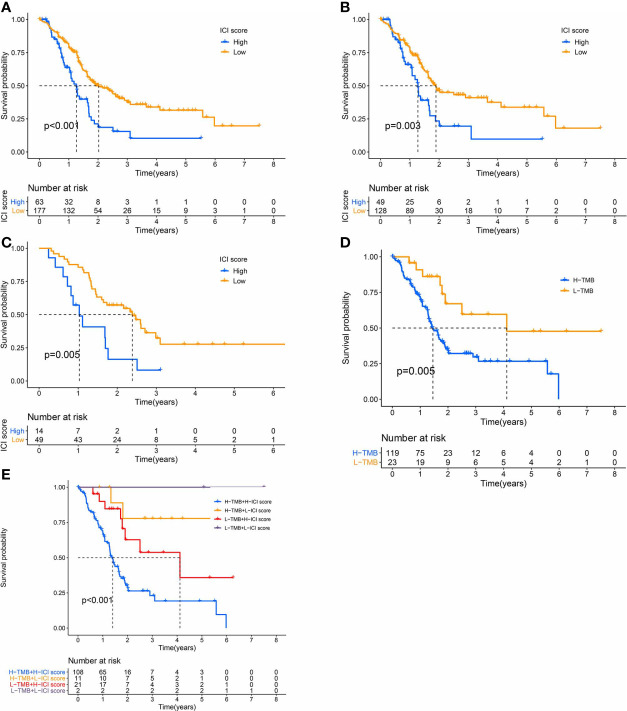
The Significance of the ICI Scores in PC prognosis. **(A-C)** Kaplan-Meier curves for high and low ICI score subgroups **(A)** for TCGA and GEO cohorts, Log-rank test, *P*<0.001; **(B)** for TCGA cohort, Log-rank test, *P*=0.003; **(C)** for GEO cohort, Log-rank test, *P*=0.005). **(D)** Kaplan-Meier curves for high and low TMB subgroups of the TCGA cohort, Log-rank test, *P*=0.005. **(E)** Kaplan-Meier curves for patients in the TCGA cohort, classified by both TMB and ICI scores, Log-rank test, *P*<0.001.

We, next, examined the prognostic capabilities of the ICI score. Using the Kaplan-Meier plot, we demonstrated that the low ICI score group had markedly improved OS rate, compared to the high ICI score group (median survival time 738 vs. 460days, log-rank test, *P *< 0.0001). Additionally, the ICI score prognosis accuracy was further confirmed in TCGA-PAAD and GEO-GSE57495, respectively (**Figures 5A–C**).

We also assigned patients to distinct groups, according to the TMB immune threshold, as described previously. Based on our analysis, patients with low TMB experienced improved OS, relative to high TMB (log-rank test, *P* = 0.005, **Figure 5D**). Given that TMB may have an impact on the prognostic capabilities of the ICI score, we next examined the collaborative impact of ICI score and TMB in predicting prognosis. The stratified survival analysis demonstrated that TMB failed to regulate ICI score-based prognosis estimation. In fact, the ICI score exhibited marked OS differences in both high and low TMB groups (log-rank test, Low TMB & Low ICI score (LL) versus Low TMB & High ICI score (LH); High TMB & Low ICI score (HL) versus High TMB & High ICI score (HH), **Figure 5E**). Collectively, these data are indicative of the predictive potential of the ICI score in PC prognosis, independent of TMB.

## Discussion

Immunotherapy has remarkable efficacy in multiple malignancies. However, only a small population of PC patients respond positively to immunotherapy, owing to the complicated TME (8). Therefore, it is urgent and necessary to explore the TME in PC patients in order to predict prognosis, design personalized therapy, and aid in the development of new PC therapies. In a previous study, researchers examined the ICI profile of PC, stratified PC patients according to infiltrating T-cell activity in TME and correlated cytolytic immune activity with mutational, structural, and neoepitope features of the tumor (31). The previous study provided insight into the intrinsic activities of PC TME. However, we aimed to explore the PC TME from a new perspective. Here, we developed a methodology for quantifying a comprehensive PC TME. Based on our analysis, we recommend the ICI score to be a promising prognostic biomarker for PC.

Different types of tumors vary widely in their TME contexture, especially in ICI (32). The TME heterogeneity, which impacts tumor progression and prognosis, has been identified in both human and murine PC (31, 33–35). PC is classified into different subtypes based on the TME heterogeneity. Moreover, an abundance of intratumoral CD8^+^ T cells is closely correlated with the response to immunotherapy in PC (33–35). However, multiple clinical and genomic studies have suggested that PC, unlike other types of cancer, is correlated with low ICI, especially CD8^+^ T cells (12, 36, 37). This may explain why only a minority of PC patients are sensitive to immunotherapy, relative to other types of tumors with high CD8^+^ T cell infiltration. In this study, we evaluated the ICI in 245 PC samples and assigned the samples to two discrete immune categories and gene clusters. We demonstrated that the levels of CD4^+^ T cells, CD8^+^ T cells, M1 macrophages, and the high immune score didn’t predict PC prognosis, unlike previous reports (38, 39). The PC TME is complex and may interfere with the intercellular interactions between infiltrating immune cells, thereby affecting immunity tolerance and activity (11, 40). Given these circumstances, the immune phenotypes and gene clusters may not serve as an appropriate biomarker to aid in predict PC prognosis and sensitivity to immunotherapy (**Figures S1A, B**).

Given the diverse nature of the immune milieu in PC patients, it was critical to characterize the ICI patterns of individual PC patients. The individual-based model, derived from tumor subtype-specific biomarkers, has been widely used in predicting prognosis in breast and colorectal cancers (41, 42). Here, using the Boruta algorithm, we identified PC biomarkers and generated an individualized ICI score to assess ICI patterns. With the help of GSEA, we also discovered three metabolic pathways (glycerolipid metabolism; fatty acid metabolism; glycine, serine and threonine metabolism) were enriched and these metabolism‐related genes were lowly expressed in the low ICI score group, relative to the high ICI score group. Emerging evidence has revealed that oncogenes can induce metabolic alterations in tumor cells and TIICs, that can restrict immune responses to cancer therapy and promote poor prognosis (43, 44). Previous studies have suggested that, in TME, competition for nutrients between cancer cells and T cells contributes to immunosuppression (45, 46). Moreover, a recent study evaluated metabolic features of tumor cell types *in vivo* and revealed that individual cell populations had distinct programs of nutrient uptake that might serve an important role in the development of future cancer therapies by altering TME (47). In addition, we, also, observed markedly elevated expression of cell cycle- and DNA repair- (mismatch repair and nucleotide excision repair) related genes in the high ICI score group. Recent studies indicated that the cell cycle activity of both cancer and immune cells in TME could mediate antitumor immunity (48–50). Meanwhile, cell cycle inhibition can result in both cell autonomous (e.g., dysregulation of the antigen presentation machinery), and non-cell autonomous (e.g., release of SASP-associated factors and T cell recruiting chemokines) mechanisms of antitumor immunity (50). This finding has strong implications for PC therapy, as it suggests that existing drugs that modulate the cell cycle, may potentially have an added (and untapped) benefit of sensitizing tumors to immunotherapy. A complex DNA repair machinery has evolved to protect genomic integrity in the face of numerous sources of DNA damage. When DNA repair fails, this damage can lead to carcinogenesis and tumor genomic instability (51). Although the coordinated activities of DNA repair pathways can quickly correct most DNA damages, delayed or improper repairs can lead to changes in the tumor genome, thereby reconstructing the TME (51, 52). A large amount of evidence shows that DNA repair plays an important role in driving sensitivity and response to immunotherapy (53, 54).

High PD-L1 levels are common in tumor cells, but not normal cells (55, 56). Several cancer types can exhibit immunosuppressed TME, along with elevated levels of PD-L1, which inhibits T-cell-mediated cytotoxicity of tumor cells (57). This may contribute to the correlation of elevated PD-L1 levels to poor prognosis. In a meta-analysis study involving PC patients, 19-62.5% of patients exhibited elevated PD-L1 levels and corresponding a poor prognosis, as opposed to those with low PD-L1 levels (58). In accordance with other studies, we also demonstrated that a high ICI score, with elevated PD-L1 levels, had a poor prognosis, relative to a low ICI score. Moreover, immunotherapies like PD-1/PD-L1 antibodies were found to be highly efficient in treating other forms of cancers but exhibited little success in PC therapy (10, 59). The specific insensitivity to immunotherapy, observed with PC patients, may be due to the PC-specific TME (10, 59). In PC, abundant extracellular matrix (ECM) surrounds tumor cells and creates a physical barrier that blocks entry of drugs and cytotoxic T cells (60). As such, PC therapy must first involve manipulation of the PC stroma to allow for the infiltration of T cells and progression of antitumor immunity. Alternately, TGF-β is involved in the pancreatic stellate cell-mediated secretion of the stiff fibrillary ECM, that sustains tumor survival (61, 62). Moreover, recently, it was suggested that TGF-β also protected tumor cells by restricting infiltrating T cells (63). Based on our analysis, TGF-β was highly expressed in the high ICI score group, which may have contributed to subsequent poor PC prognosis and immunotherapy failure. Single-agent immunotherapies are therefore ineffective in PC. Several reports have suggested that in tumors with low T cell infiltration, the combined blockade of the PD-L1 checkpoint and TGF-β signaling pathway can enhance CD8^+^ T cell infiltration in TME and stimulate strong anti-tumor immunity (64, 65). As such, the synergistic suppression of TGF-β and PD-L1 pathways may be an effective therapy to curb PC.

In addition, in most tumors, a high mutation load is equivalent to an increased amount of tumor antigens, which can regulate survival benefits by reshaping the TME, and can also be used as a biomarker for immunotherapy responsiveness and prognosis (66, 67). In terms of mechanism, high TMB provides more opportunities for the generation of “non-self” neoantigens, which can activate the enrichment of immune cells (68). However, these theories have only been confirmed in some immunotherapeutic hot tumors. However, in immunotherapeutic cold tumors, such as PC, these rules may not apply. PC is known to have a low tumor mutation load, which may contribute to the unresponsiveness of PC immunotherapy (69). Based on our analysis, the PC patients with a low mutation load had a better prognosis. This may be attributed to the unique population of dysfunctional antigen-presenting dendritic cells in PC, which failed to induce anti-cancer immunity. Instead, the Th17 cells released IL-17 to modulate TME in order to promote tumor growth and metastasis (70). Based on our analysis, the association between the ICI score and TMB was -0.25. Using stratified analysis, we demonstrated that the prognosis, using the ICI score, was not regulated by TMB in PC. Furthermore, the absence of an ICI score-TMB correlation is indicative of these factors modulating individual aspects of tumor immunobiology. In our analysis, the ICI score was found to be predictive of PC patient prognosis, independent of TMB.

There were several limitations to our study. First, the available public dataset for PC is limited. So, in this study 245 tumor samples were obtained from the TCGA-PAAD and GEO-GSE57495 cohorts. Hence, the sample size might be relatively small. A larger sample population will aid in a better understanding of the PC ICI profile. Second, we performed bioinformatic analysis of PC samples and developed an index, the ICI score, to stratify patients. Specifically, the ICI score may serve as a tool for the classification of PC patients and may contribute to the clinical treatment of patients. Some expressions of immune-related genes and enrichments of molecular pathways were different between the ICI score subgroups. The subgroup-specific differences could also serve as potential therapeutic targets. Further *in vitro* and *in vivo* validations will be helpful to discover these potential therapeutic targets.

## Conclusion

In conclusion, we extensively evaluated the ICI profile in PC patients, enabling a clearer understanding of the anti-/pro-tumor immune response in PC. According to our analysis, the ICI score can serve as a valid prognostic biomarker of PC. Therefore, a systematic assessment of tumor ICI patterns in PC patients is essential to individualized PC therapy.

## Data Availability Statement

Publicly available datasets were analyzed in this study. This data can be found here: TCGA at https://portal.gdc.cancer.gov, reference number TCGA-PAAD, and GEO at https://www.ncbi.nlm.nih.gov/geo/, under the accession number GSE57495.

## Author Contributions

SS and DL conceived and designed the whole project. DL and FK analyzed the data and wrote the manuscript. JD and MZ carried out data interpretations. FP, YG, and CF provided specialized expertise and collaboration in data analysis. BL and XY revised the manuscript. All authors contributed to the article and approved the submitted version.

## Funding

This work was supported by the Natural Science Youth Fund of Southwest Medical University [Grant Number: 2019ZQN072].

## Conflict of Interest

The authors declare that the research was conducted in the absence of any commercial or financial relationships that could be construed as a potential conflict of interest.

## Supplementary Material

Supplementary Figure 1
**(A)** Kaplan-Meier curves for the discrete ICI clusters of the TCGA and GEO cohorts, Log-rank test, *P*=0.995. **(B)** Kaplan-Meier curves for the discrete gene clusters of the TCGA and GEO cohorts, Log-rank test, *P*=0.256.
